# Alcohol pictorial health warning labels: the impact of self-affirmation and health warning severity

**DOI:** 10.1186/s12889-018-6243-6

**Published:** 2018-12-22

**Authors:** Carlos Sillero-Rejon, Angela S. Attwood, Anna K. M. Blackwell, José-Angel Ibáñez-Zapata, Marcus R. Munafò, Olivia M. Maynard

**Affiliations:** 1 UK Centre for Tobacco and Alcohol Studies, School of Psychological Science, University of Bristol, Bristol, UK; 2 MRC Integrative Epidemiology Unit (IEU) at the University of Bristol, Bristol, UK; 3 Department of Marketing and Market Research, University of Granada, Granada, Spain; 4 School of Psychological Science, University of Bristol, 12a Priory Road, Bristol, BS8 1TU UK

**Keywords:** Alcohol, Eye-tracking, Self-affirmation, Severity, Health-warning

## Abstract

**Background:**

We examined whether enhancing self-affirmation among a population of drinkers, prior to viewing threatening alcohol pictorial health warning labels, would reduce defensive reactions and promote reactions related to behaviour change. We also examined how health warning severity influences these reactions and whether there is an interaction between self-affirmation and severity.

**Methods:**

In this experimental human laboratory study, participants (*n* = 128) were randomised to a self-affirmation or control group. After the self-affirmation manipulation was administered, we tracked participants’ eye movements while they viewed images of six moderately-severe and six highly-severe pictorial health warning labels presented on large beer cans. Self-reported responses to the pictorial health warning labels were then measured, including avoidance, reactance, effectiveness, susceptibility and motivation to drink less. Finally, participants reported their self-efficacy to drink less and their alcohol use.

**Results:**

There was no clear evidence that enhancing self-affirmation influenced any outcome. In comparison to moderately-severe health warnings, highly-severe health warnings increased avoidance and reactance and were perceived as more effective and increased motivation to drink less.

**Conclusions:**

These findings call into question the validity of the self-affirmation manipulation, which is purported to reduce defensive reactions to threatening warnings. We discuss possible explanations for this null effect, including the impact of participants’ low perceived susceptibility to the risks shown on these pictorial health warning labels. Our finding that highly-severe health warnings increase avoidance and reactance but are also perceived as being more effective and more likely to motivate people to drink less will inform future health warning design and have implications for health warning label theory.

**Electronic supplementary material:**

The online version of this article (10.1186/s12889-018-6243-6) contains supplementary material, which is available to authorized users.

## Background

Harmful use of alcohol causes more than 3.3 million deaths every year, accounting for almost 6% of all global deaths [1]. Moreover, alcohol is the third leading cause of morbidity and mortality in the European Union (EU), and Europe is the world’s heaviest drinking region [2]. While tobacco health warning labels have been shown to affect consumer behaviour [3] and communicate risk [4], similar labels are not widely applied to alcohol products. It has been suggested that similar health warnings be placed on alcohol products [2, 5, 6], particularly given the low cost of implementation [7] and high levels of public support [6, 8, 9].

In 2011, alcohol companies in the United Kingdom (UK) pledged to put health warnings on 80% of alcohol containers as part of the Responsibility Deal, although this pledge has not been fully met [10]. Eye-tracking research by Kersbergen and Field [11] suggests that consumers pay minimal attention to these voluntary warnings and they have no impact on drinking intentions. We have observed similar effects among daily smokers who actively avoid pictorial health warnings on cigarette packs [12]. Consumers have low awareness of the current voluntary warnings on alcohol containers and are unable to recall and recognise them [13].

To improve the efficacy of alcohol health warnings, research suggests that warnings should incorporate images as well as text, and that the images should be pictorial. To increase their salience [2, 14], they should be in a standard location, parallel to the base of the container and separate from other label information [2], and they should cover a set minimum size of the product label [15]. Regarding the messages themselves, it has been suggested they use a serious tone, with simple, clear and unambiguous language [6]. The messages should increase perceived threat, both by increasing perceived susceptibility to the risk presented and by demonstrating the severity of the risk [16].

However, the Extended Parallel Process Model (EPPM) suggests that health warnings which only increase threat, but do not increase an individuals’ efficacy to deal with a threat, are likely to lead to defensive reactions [16] which may compromise warning’s effectiveness. These defensive reactions include avoiding warnings as a coping mechanism (i.e., avoidance) [12] or the opposition to them if they are perceived as a threat to one’s autonomy (i.e., reactance) [17]. This theory suggests that defensive reactions to health warnings may be enhanced by increasing the severity of the message presented and evidence indicates that highly-severe health warnings are poorly recalled [18]. However, it has been also observed that highly-severe health warnings increase intention to quit [18] and are perceived as more effective than moderately-severe health warnings [19].

Highly-severe health warnings may increase defensive reactions because they are seen as damaging to consumers’ self-view [20]. By restoring one’s global positive self-image from threats, self-affirmation manipulations (i.e., tasks which increase an individual’s self-image) may be a method of reducing the psychological discomfort experienced as a result of being exposed to health warnings, thereby reducing defensive reactions to them [21]. Previous research has found that self-affirmation manipulations encourage less defensive responses to threatening communications for a range of health behaviours [22–25] including alcohol consumption [26–30].

The main aim of this study is to examine whether enhancing self-affirmation among drinkers prior to viewing alcohol pictorial health warning labels reduces defensive reactions and promotes positive responses to these warnings. We hypothesise that compared to non-self-affirmed participants, self-affirmed participants will report less avoidance and reactance to warnings; but increased visual attention to warnings, perceived susceptibility to health risks, perceived effectiveness of warnings, motivation to reduce alcohol consumption and self-efficacy to drink less. Secondary aims include understanding how warning severity (i.e., moderately-severe versus highly-severe) influences these reactions. We hypothesise that participants will respond more defensively to highly-severe warnings (less visual attention, more avoidance and reactance) than to moderately-severe warnings; yet they will find highly-severe warnings to be more effective, increase perceived susceptibility and enhance motivation to reduce alcohol consumption compared to moderately-severe warnings. Finally, we hypothesise that self-affirmed participants will react less defensively to highly-severe warnings than non-self-affirmed participants.

## Methods

The protocol for this study was preregistered on the Open Science Framework: osf.io/x7vps/. Ethics approval was obtained from the Faculty of Science Research Ethics Committee at the University of Bristol (Approval Code: 50761).

### Design

This was an experimental human laboratory study using eye-tracking to measure visual attention, with a between-subjects factor of self-affirmation (self-affirmed vs. control) and a within-subject factor of warning severity (moderately-severe warnings vs. highly-severe warnings) to assess defensive and positive responses to pictorial health warnings. This was a pseudorandomized experiment: equal numbers of males and females were allocated to the two groups.

### Participants

Participants were recruited from staff and students at the University of Bristol, and members of the public, via existing email lists, word of mouth, posters and flyers and the Tobacco and Alcohol Research Group (TARG) website. Participants were required to be aged 18 or over and be regular alcohol consumers who have consumed over the UK weekly guidelines (14 units per week; equivalent to six pints of 4% beer or six 175 ml glasses of 13% wine) during the preceding week. Eligibility was assessed in an online screening form prior to recruitment into the experiment. For the power calculation, we considered the effect size of our outcome measures in previous studies [24, 31]. To detect an effect size of *d* = 0.50 for our primary outcome measure (visual attention to the warnings), we required 128 participants (64 per self-affirmation condition) to achieve 80% power at an alpha level of 5%.

### Materials and measures

#### Stimuli

**Fig. 1:**
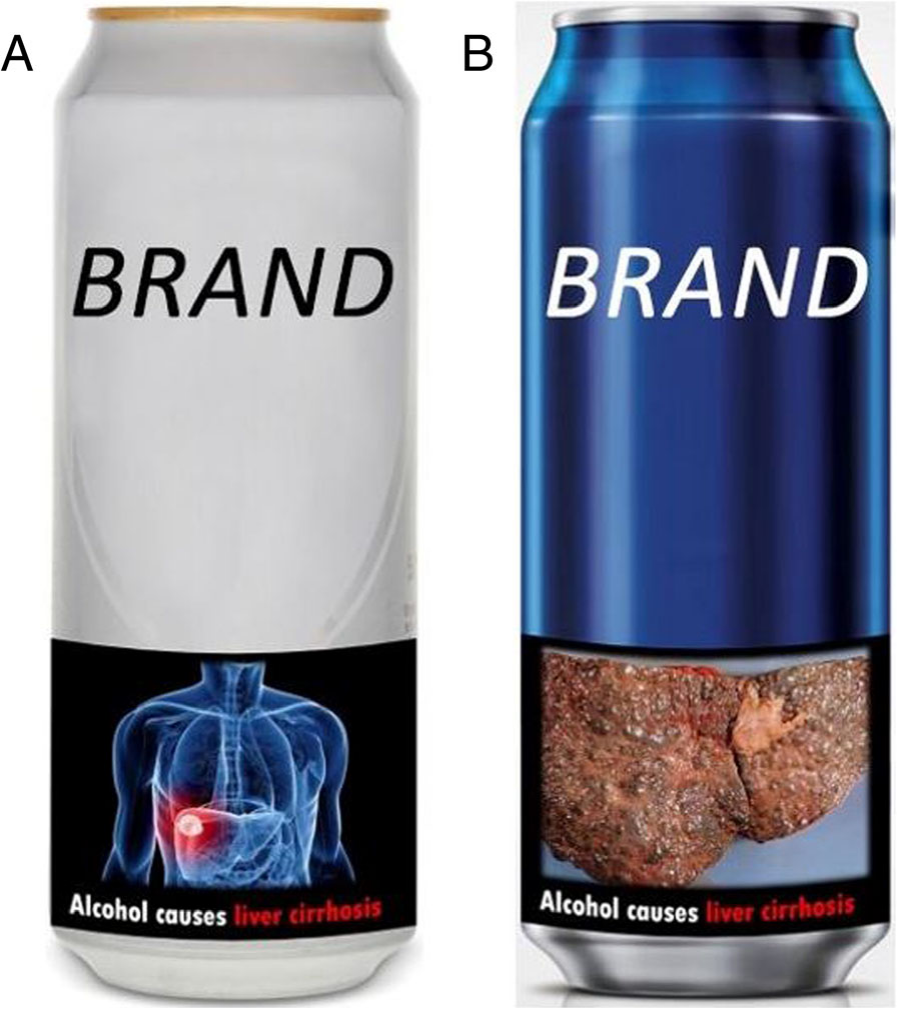
Stimuli Examples. Examples of stimuli shown to participants during which their eye movements were recorded. “**a**” show as an example of a moderately severe graphic health warning for liver cirrhosis. “**b**” shows an example of a highly severe graphic health warning for liver cirrhosis. Brands have been removed in these stimuli examples. Images source: composition; study authors. Liver cirrhosis warnings: moderately severe; source: 123RF Europe BV; **©** 123RF Europe BV/Sebastian Kaulitzki; Permission acquired. Highly severe; source: Medscape

Alcohol stimuli were images of large cans of beer (presented as 379 × 675 pixels) from the most popular brands in the UK based on market share data (The Grocer, 2016). Six moderately-severe and six highly-severe pictorial health warnings were presented on the bottom third of the cans. A pilot study was conducted to assess the warnings’ consistency, realism and severity (data can be found in the preregistered protocol). Following the recommendations of Thomson, Vandenberg [6] and Eurocare [2], the health outcomes were: liver cirrhosis, brain damage, mental illness, cancer, road accidents and risk to an unborn child. Impotence was excluded because of the difficulty in creating moderately-severe and highly-severe warnings. Figure 1 shows some examples of the stimuli used (with the branding removed for the purposes of publication).

#### Self-affirmation manipulation

The value essay is a standard manipulation for promoting self-affirmation [32]. Although there are a number of different procedures, we followed the procedure used by Klein and Harris [29]. Participants were provided with a list of values and those in the self-affirmation group were asked to select their most important value and write a short essay about why it was important to them. Non-self-affirmed participants (control group) were asked to choose their least important value and write about why it could be important for someone else. Equal numbers of participants were randomly allocated to the self-affirmation and control group with gender counterbalanced.

#### Visual attention

The primary outcome was the number of fixations towards the pictorial health warnings. The secondary outcome measures were the total duration of fixations towards the warnings and the number of times that the first fixation was located on the warnings.

#### Avoidance and reactance

A subset of avoidance questions was taken and adapted from the PATH [33] . The questions: ‘*How likely is it that you would try to avoid thinking about the warning?*’, ‘*How likely is it that you would try to avoid looking at the warning on your alcohol can?*’, ‘*How likely is it that you would keep the can out of sight to avoid looking at the warning?*’ were answered on a five-point scale from *‘not at all likely’* (coded as 1) to *‘extremely likely’* (coded as 5). Reactance to the warning was assessed via the Brief Reactance to Health Warning Scales [34]. Agreement to the statements: ‘*This warning annoys me*’, ‘*This warning is trying to manipulate me’* and ‘*The health effect on this warning is overblown’* was scored on a five-point scale from ‘*strongly disagree*’ (coded as 1) to ‘*strongly agree*’ (coded as 5). We calculated mean avoidance and reactance scores from each pictorial health warning rating.

#### Susceptibility

Perceived susceptibility to the health risks was measured using the one-item scale adapted from Witte [16], previously used in the literature [22, 35]: ‘*How likely is it that I will experience the problems described in the message if I do not change my drinking behaviour*’. Participants rated each pictorial health warning individually using a numeric scale, where 1 was ‘*not at all likely’* and 5 *‘very likely’*.

#### Effectiveness

Perceived warning effectiveness was measured using questions designed to measure tobacco warning effectiveness [36, 37]. Participants were asked an overall effectiveness question: ‘*How effective is this health warning?*’ Participants were asked to rate each warning individually using a numeric scale, where 1 is ‘*not at all*’ and 5 ‘*extremely*’.

#### Motivation

The impact of each warning on motivation to drink less was assessed using the following question adapted from Fathelrahman, Omar [38] and Noar, Hall [4]: ‘*To what extent would this warning motivate you to drink less?*’ rated from 1 (‘*not at all*’) to 5 (‘*a lot’*).

#### Self-efficacy

Perceived self-efficacy to drink less was measured using the items: ‘*Overall, how confident are you that you can stop drinking altogether right now?*’ (‘*not at all*’, ‘*slightly’*, ‘*somewhat’*, ‘*very’*, and ‘*completely confident*’) and ‘*For me cutting down on the number of alcohol units that I drink in the next week would be…*’ (‘*very difficult* – ‘*very easy*’). Items were rated from one to five. This scale was an adaption of one used by Harris, Mayle [24]. Total self-efficacy was calculated as the mean of these two items.

#### Alcohol consumption and use

Alcohol consumption was assessed using two measures. For screening, we used a retrospective 7-day alcohol recall questionnaire where participants selected the number of drinks that they had in the preceding week; a wide range of options was provided (e.g., pint of 4% beer, pint of 5% cider, 175 ml glass of 13% wine or 25 ml shot of 40% spirit). Participants could also answer using a free-text option if the drinks they consumed were not shown in the questionnaire.

During the experiment we also took the total score from the Alcohol Use Disorders Identification Test (AUDIT) [39], which contains a 10-item questionnaire developed by the World Health Organization to asses alcohol consumption, drinking behaviour and alcohol related problems.

### Procedure

Interested participants completed an online questionnaire through the web application Qualtrics (http://www.qualtrics.com/). This survey included eligibility questions, including the retrospective alcohol recall questionnaire.

Eligible participants were invited to attend a single testing session lasting approximately 45 min. Once participants were in the lab, they provided informed consent and were allocated to either the self-affirmation or control group using a double blinded pseudorandomized design. Participants either completed the self-affirmation task or the control task through a computer-based questionnaire. Immediately afterwards, participants completed the eye-tracking task. Participants were sat 57 cm from an LCD computer screen and were fitted with an Eyelink II eye tracker (SR Research Ltd., ON, Canada) to measure their eye movements after a 9-point calibration task. Across four blocks, participants viewed 24 alcohol cans each with an alcohol pictorial health warning; each block included six different cans of beer and six different warnings. Each image was presented individually on screen for 10,000 ms and each warning was presented twice during the task. In order to promote attention during the eye-tracking task, participants completed a recall task at the end of each block, which involved identifying whether the images presented were shown in the previous block or not (this data was not analysed). Immediately after completing the eye-tracking task, participants were provided with an online questionnaire to assess self-efficacy, and the 12 warnings were individually and randomly presented accompanied by the rest of the measures, also presented in a randomised order. Finally, participants answered the AUDIT questionnaire. Participants were then fully debriefed and reimbursed £7 or granted course credits. Further details can be found in the preregistered protocol (https://osf.io/qtyha/).

### Statistical analysis

To answer the question as to whether self-affirmation and/or warning severity influences visual attention, we conducted a 2 (self-affirmation: self-affirmed vs. control) × 2 (warning severity: moderately-severe vs. highly-severe) mixed model MANOVA for the visual attention measures (i.e., number of fixations, durations of fixations and times that the first fixation was located on the warnings). A similar MANOVA model was performed for self-reported measures (i.e., avoidance, reactance, susceptibility, effectiveness and motivation). Finally, we used a one-way ANOVA to examine whether overall self-efficacy to drink less was different among those in the self-affirmation condition as compared with those in the control condition.

We conducted a planned exploratory analysis (i.e., in our pre-registered protocol) to describe the differences (means and standard deviations) in the outcome measures between the six different health outcomes shown in the warnings.

Additional unplanned analyses (i.e., not in our pre-registered protocol) were performed to explore the effect of self-affirmation between groups with different self-efficacy levels. We classified participants according to their level of self-efficacy using a hierarchical cluster analysis (which provided us with the number of clusters) to run in a k-means cluster analysis. Given the unplanned nature of these analyses and the lack of a pre-defined hypotheses, we have not conducted statistical tests and instead we present here estimated marginal means (EMM), standard errors (SE) and confidence intervals (CI).

## Results

The data that forms the basis of the results presented here are available from the University of Bristol Research Data Repository (http://data.bris.ac.uk/data/), doi: 10.5523/bris.2iopqddd82mvx2sa0i0lqx87jt. For visual attention model, univariate results for duration of fixations and the times that the first fixation was located on the pictorial health warnings are reported in the Additional file 1.

### Characteristics of participants

Participants were 128 regular alcohol drinkers; 64 per experimental group with equal numbers of females and males. The mean age was 22 (*SD* = 4). Participants reported having drunk on average 34 (*SD* = 17) alcohol units in the week prior to signing up for the experiment, equivalent to 15 pints of 4% beer or 15, 175 ml glasses of 13% wine. The average AUDIT score was 14 (*SD* = 5), which is towards the upper limit of Zone II which suggests a medium level of alcohol problems [40].

### Self-affirmation

On average, participants spent 47% of the time looking at the pictorial health warnings.

The multivariate results for visual attention showed no clear evidence for self-affirmation on the combined dependent variables (Wilks’ Lambda = 0.99, *F*(3,124) = 0.25, *p* = 0.86, *η*_p_^2^ = 0.006). The univariate tests indicated that there was no clear evidence that self-affirmation manipulation had an effect on number of fixations (*F*(1,128) = 0.035, *p* = 0.52, *η*_p_^2^ < 0.001). Table 1 shows descriptive statistics for number of fixations.

**Table 1:** Visual attention

Group	Number of fixations	
	Highly Severe	Moderately Severe
Control (*n* = 64)	16.31 (4.53)	16.07 (4.78)
Self-Affirmed (*n* = 64)	16.48 (5.22)	16.23 (5.08)
Mean (*n* = 128)	16.40 (4.87)	16.15 (4.92)

Means and standard deviations (in parentheses) for number of fixations toward pictorial health warnings by self-affirmation condition (self-affirmation condition vs. control condition) and for each health warning severity condition (highly-severe vs. moderately-severe pictorial health warning)

The multivariate results for self-reported measures showed no clear evidence for self-affirmation on the combined dependent variables (Wilks’ Lambda = 0.96, *F*(5,122) = 0.96, *p* = 0.45, *η*_p_^2^ = 0.04). When results for dependent variables were considered separately, there was no clear evidence that self-affirmation manipulation had an effect on any of the outcome measures: avoidance (*F*(1,128) = 0.18, *p* = 0.67, *η*_p_^2^ < 0.001), reactance (*F*(1,128) = 0.33, *p* = 0.57, *η*_p_^2^ < 0.001), susceptibility (*F*(1,128) = 0.01, *p* = 0.92, *η*_p_^2^ < 0.001), effectiveness (*F*(1,128) = 0.4, *p* = 0.84, *η*_p_^2^ < 0.001) or motivation to drink less (*F*(1,128) = 1.38, *p* = 0.24, η_p_^2^ = 0.01). Finally, there was also no clear evidence that self-affirmation influenced self-efficacy to drink less (*F*(1,128) = 1.02, *p* = 0.32). Table 2 shows descriptive statistics for self-reported measures.

**Table 2:** Self-reported measures

	Avoidance		Reactance		Susceptibility		Effectiveness		Motivation		Self-efficacy
Group	Highly Severe	Moderately Severe	Highly Severe	Moderately Severe	Highly Severe	Moderately Severe	Highly Severe	Moderately Severe	Highly Severe	Moderately Severe	
Control (*n* = 64)	3.33 (0.92)	2.30 (0.84)	2.39 (0.82)	2.16 (0.71)	2.05 (0.59)	2.07 (0.52)	3.26 (0.65)	2.50 (0.64)	2.53 (0.64)	2.14 (0.68)	3.11 (0.99)
Self-Affirmed (*n* = 64)	3.19 (0.89)	2.32 (0.71)	2.46 (0.75)	2.23 (0.58)	2.10 (0.69)	2.05 (0.63)	3.23 (0.69)	2.49 (0.53)	2.73 (0.79)	2.20 (0.63)	3.30 (1.11)
Mean (*n* = 128)	3.26 (0.91)	2.31 (0.77)	2.42 (0.78)	2.20 (0.65)	2.08 (0.64)	2.06 (0.57)	3.25 (0.67)	2.50 (0.58)	2.63 (0.72)	2.17 (0.65)	3.20 (1.05)

Means and standard deviations (in parentheses) for self-reported scores for avoidance, reactance, susceptibility, effectiveness and motivation by self-affirmation condition (self-affirmation condition vs. control condition) and for each health warning severity condition (highly-severe vs. moderately-severe pictorial health warning)

### Severity

The multivariate results for visual attention showed no clear evidence for warning severity on the combined dependant variables (Wilks’ Lambda = 0.97, *F*(3,124) = 1.33, *p* = 0.27, *η*_p_^2^ = 0.03). The univariate tests showed that there was no clear evidence of an effect of warning severity on number of fixations (*F*(1,128) = 1.90, *p* = 0.17, *η*_p_^2^ = 0.02). Table 1 shows descriptive statistics for number of fixations.

The multivariate results for self-reported measures showed evidence for warning severity on the combined dependant variables (Wilks’ Lambda = 0.27, *F*(5,122) = 0.035, *p* < 0.001, *η*_p_^2^ = 0.72). When dependant variables were considered separately, the univariate results indicated that compared with moderately-severe warnings, highly-severe warnings received higher scores for self-reported avoidance (*F*(1,128) = 260.84, *p* < 0.001, *η*_p_^2^ = 0.67), reactance (*F*(1,128) = 32.99, *p* < 0.001, *η*_p_^2^ = 0.21), effectiveness (*F*(1,128) = 137.59, *p* < 0.001, *η*_p_^2^ = 0.52) and motivation to drink less (*F*(1,128) = 83.80, *p* < 0.001, *η*_p_^2^ = 0.4). However, warning severity did not appear to impact perceived susceptibility to the health risk shown on the warning (*F*(1,128) = 0.23, *p* = 0.63, *η*_p_^2^ < 0.001). Descriptive statistics are shown in Table 2.

### Interaction between self-affirmation and severity

The multivariate results for visual attention showed no clear evidence of an interaction effect between warning severity and self-affirmation on the combined dependent variables (Wilks’ Lambda = 0.96, *F*(3,124) = 1.75, *p* = 0.16, *η*_p_^2^ = 0.04). The univariate tests indicated no clear evidence for this interaction effect for number of fixations (*F*(1,128) = 1.90, *p* = 0.17, *η*_p_^2^ < 0.001). Table 1 shows descriptive statistics for number of fixations.

The multivariate results for self-reported measures showed no clear evidence of an interaction effect between warning severity and self-affirmation on the combined dependent variables (Wilks’ Lambda = 0.93, *F*(5,122) = 1.73, *p* = 0.13, *η*_p_^2^ = 0.07). The univariate tests showed this lack of evidence for all of the outcome measures: avoidance (*F*(1,128) = 1.92, *p =* 0.17, *η*_p_^2^ = 0.01), reactance (*F*(1,128) = 0.003, *p* = 0.96, *η*_p_^2^ < 0.001), susceptibility (*F*(1,128) = 0.93, *p* = 0.34, *η*_p_^2^ < 0.001), effectiveness (*F*(1,128) = 0.01, *p* = 0.90, *η*_p_^2^ < 0.001) and motivation to drink less (*F*(1,128) = 2.12, *p* = 0.15, *η*_p_^2^ = 0.02). Descriptive statistics are shown in Table 2.

### Planned exploratory analyses

In the Additional file 1, we report the differences in the number of fixations and self-reported measures of avoidance, reactance, susceptibility, effectiveness and motivation to drink less for the six different health outcomes shown in the warnings: liver cirrhosis, brain damage, mental illness, cancer, road accidents and risk to an unborn child.

### Unplanned exploratory analyses

In unplanned analyses, we classified participants into two clusters: high self-efficacy (*n* = 60, *M* = 4.18, *SD* = 0.47) and low self-efficacy (*n* = 68, *M* = 2.33, *SD* = 0.52). We found that among those in the high self-efficacy cluster, those who were self-affirmed reported lower levels of avoidance (*EMM* = 2.70, *SE* = 0.13, *CI* = 2.43,2.96) than those in the control condition (*EMM* = 3.11, *SE* = 0.14, *CI* = 2.82,3.39). Conversely, among those in the low self-efficacy cluster, those who were self-affirmed reported higher levels of avoidance (*EMM* = 2.81, *SE* = 0.13, *CI* = 2.55,3.08) than those in the control condition (*EMM* = 2.58, *SE* = 0.13, *CI* = 2.33,2.84).

## Discussion

This study examined whether enhancing self-affirmation among a group of drinkers, prior to viewing pictorial health warnings on beer containers, reduces defensive reactions and promotes positive reactions We did not find evidence that enhancing self-affirmation among drinkers impacted any of our measures of negative and positive reactions. Nevertheless, in unplanned analyses we found some evidence that among those participants with higher self-efficacy to reduce drinking, self-affirmation may reduce avoidance to warnings. We also investigated how warning severity (moderately-severe versus highly-severe) influences these reactions, and whether there is an interaction between self-affirmation and warning severity. We found no clear evidence for a difference regarding visual attention to moderately-severe versus highly-severe warnings; however, there was more avoidance and reactance to highly-severe warnings, which were also perceived as more effective and promoted higher motivations to reduce drinking. Finally, we did not find evidence for an interaction between self-affirmation and warning severity.

Contrary to previous research that reports positive effects of self-affirmation on drinking behaviour [26–30], we found no effect of self-affirmation on any of our outcome measures. Our results are in line, however, with previous research which found no effect of self-affirmation on fixations to tobacco pictorial health warnings [31]. Our sample’s low levels of self-reported perceived susceptibility towards the health risks shown in the warnings may explain these null effects. Self-affirmation aims to restore one’s positive self-image from threats [21] by reducing the psychological discomfort caused by cognitive dissonance [41]. If participants did not experience any psychological discomfort from the warnings, as they did not consider themselves susceptible to the risks described, the warnings would not have posed a threat to their self-image and the self-affirmation manipulation would arguably have no effect. On the other hand, through an unplanned analysis, we found some evidence to suggest that for individuals with high levels of self-efficacy, self-affirmation may reduce negative reactions to warnings. This suggests that self-affirmation may play a role in reducing the psychological discomfort from the threat of warnings among those with higher levels of self-efficacy (i.e., if individuals believe that they have the ability to change the risky behaviour). This suggests that there may be a role for self-affirmation interventions in high self-efficacy groups and that combining self-affirmation with self-efficacy interventions in other groups may be beneficial. Future research should explore this possibility. However, these unplanned exploratory results must be interpreted with caution.

Although we did not find any evidence of a difference in visual attention to highly-severe warnings and moderately-severe warnings, we found that severity increases avoidance and reactance to these warnings. This difference between the methodologies is unsurprising, given that eye-tracking (i.e., visual attention outcome) is an objective measure, while self-reported avoidance and reactance are subjective measures of the effectiveness of communicating threat [3]. These findings replicate our previous results with moderately- and highly-severe tobacco pictorial health warnings (Sillero-Rejon C., Leonards U., Hoek J., Toll B., Hedge C., Gove H., et al. The impact of health warning location, framing, immediacy and severity, on visual attention and self-reported avoidance. In Preparation. 2018) and align with previous literature that finds that highly-severe warnings increase perceived effectiveness and motivation to reduce drinking as compared with moderately-severe warnings [18, 19]. We did not find any evidence that there was a difference in perceived susceptibility to the risks shown between the highly-severe and moderately-severe warnings.

Our study has several limitations. First, although we recruited participants who drank above the low-risk guidelines and reported relatively high scores on AUDIT (and therefore are arguably susceptible to the risks of their alcohol consumption), we found that our participants did not perceive themselves as at risk from drinking. This may be because our participants were relatively young. Second, we only examined participants’ self-efficacy to reduce their drinking in our unplanned exploratory analyses and therefore these results must be interpreted with caution. It is possible that self-affirmation may only be effective among those individuals already with a high level of self-efficacy and future research should examine this. As discussed above, these aspects may have compromised the potential impact of the self-affirmation manipulation. Finally, despite the important strengths of eye-tracking for objectively measuring visual attention, the lack of natural environment may have impacted how our participants viewed the warnings.

Notwithstanding these limitations, this study has considerable implications. Our results call into question the utility of self-affirmation as a method to increase engagement with pictorial health warnings, particularly to increase visual attention. Regarding alcohol policy, considering that the European Commission has called for alcohol labelling on alcohol products [42], our research suggests that highly-severe content might increase engagement, particularly motivation to reduce drinking. Additionally, our finding that participants reported drinking heavily and not high perceived susceptibility to alcohol related health risks suggests that increasing this susceptibility could be an important target for encouraging changes in attitudes and ultimately behaviour.

## Conclusions

We did not find that self-affirmation influences any of our outcome measures. However, we found higher levels of self-reported engagement with highly-severe pictorial health warnings in comparison to moderately-severe pictorial health warnings. Highly-severe pictorial health warnings were rated as more effective, leading to greater motivation to reduce drinking, as well as greater avoidance and reactance.

## Additional file

Additional file 1: **Table S1.** Result for visual attention measures. **Table S2.** Univariate results for visual attention model. **Table S3.** Result for the planned exploratory analysis. (DOCX 21 kb)

## Abbreviations

AUDIT: Alcohol Use Disorders Identification.
CI: Confidence Interval
EMM: Estimated Marginal Mean
EU: European Union
M: Mean
SD: Standard Deviations
SE: Standard Errors
UK: United Kingdom
