# Supplementary material for: Alcohol pictorial health warning labels: the impact of self-affirmation and health warning severity
Source: BMC Public Health. 2018 Dec 22;18:1403. doi: 10.1186/s12889-018-6243-6 (PMC6303887; doi:10.1186/s12889-018-6243-6)
Supplement: Supplementary file 1 — Table S1. Result for visual attention measures. Table S2. Univariate results for visual attention model. Table S3. Result for the planned exploratory analysis. (DOCX 21 kb) [file 12889_2018_6243_MOESM1_ESM.docx]

**Table S1. Result for visual attention measures.**

|  | **Number of fixations** | | **Durations of fixations** | | **First fixations** | |
| --- | --- | --- | --- | --- | --- | --- |
| **Group** | **Highly severe** | **Moderately Severe** | **Highly severe** | **Moderately Severe** | **Highly severe** | **Moderately Severe** |
| **Control**  (n = 64) | 16.31 (4.53) | 16.07 (4.78) | 4720.27 (1475) | 4657.86 (1429.83) | 1.67 (2.07) | 1.52(2.12) |
| **Self-Affirmed**  (n = 64) | 16.48 (5.22) | 16.23 (5.08) | 4716.78 (1560.29) | 4573.31 (1540.57) | 1.54 (2.13) | 1.93 (2.33) |
| **Mean**  (n = 128) | 16.40 (4.87) | 16.15 (4.92) | 4718.53 (1512.26) | 4615.58 (1480.98) | 1.61 (2.09) | 1.72 (2.23) |

Means and standard deviations (in parenthesis) for visual attention measures (i.e. number of fixations, durations of fixations and the times that the first fixation was located on the pictorial health warnings) by self-affirmation condition (self-affirmation condition vs. control condition) and for each warning severity condition (highly-severe vs. moderately-severe pictorial health warning).

**Table S2. Univariate results for visual attention model.**

| **Variable/Factor** | **Self-affirmation** | **Severity** | **Interaction effect** |
| --- | --- | --- | --- |
| **Number of fixations** | *F*(1,128)=0.035, *p*=0.52, *η*_p_^2^<0.001 | *F*(1,128)=1.90, *p*=0.17, *η*_p_^2^=0.02 | *F*(1,128)=1.90, *p*=0.17, *η*_p_^2^<0.001 |
| **Durations of fixations** | *F*(1,128)=0.03, *p*=0.87, *η*_p_^2^<0.001 | *F*(1,128)=3.61, *p*=0.06, *η*_p_^2^=0.03 | *F*(1,128)=1.90, *p*=0.17, *η*_p_^2^<0.001 |
| **First Fixations** | *F*(1,128)=0.15, *p*=0.7, *η*_p_^2^=0.001 | *F*(1,128)=0.92, *p*=0.34, *η*_p_^2^=0.007 | *F*(1,128)=4.99, *p*=0.03, *η*_p_^2^=0.04 |

Univariate results from the Multivariate Analysis of Variance (MANOVA) for visual attention measures (i.e. numbers of fixations, durations of fixations and the times that the first fixation was located on the pictorial health warnings) with self-affirmations as a between subject factor, health warning severity as within subject factor and the interaction effect between these factors.

**Table S3**. **Results for the planned exploratory analysis.**

| **Health risk** | **Number of fixations** | **Avoidance** | **Reactance** | **Susceptibility** | **Effectiveness** | **Motivation** |
| --- | --- | --- | --- | --- | --- | --- |
| **Road Accidents** | 18.35 (5.61) | 2.39 (0.91) | 2.02 (0.75) | 1.64 (0.75) | 2.82 (0.78) | 2.18 (1.03) |
| **Cirrhosis** | 17.07 (6.05) | 2.93 (0.90) | 2.20 (0.74) | 2.54 (0.89) | 2.87 (0.76) | 2.60 (0.82) |
| **Mental Health** | 16.10 (4.60) | 2.78 (0.98) | 2.55 (0.90) | 2.18 (0.96) | 2.80 (0.80) | 2.39 (0.90) |
| **Cancer** | 15.71 (5.38) | 3.05 (1.00) | 2.52 (0.90) | 2.27 (0.79) | 2.84 (0.84) | 2.54 (0.83) |
| **Pregnancy** | 15.56 (5.24) | 2.82 (0.94) | 2.09 (0.82) | 1.57 (0.90) | 3.23 (0.96) | 2.24 (1.20) |
| **Brain Damage** | 14.84 (4.78) | 2.73 (0.81) | 2.48 (0.79) | 2.21 (0.78) | 2.68 (0.72) | 2.44 (0.80) |

Means and standard deviations (in parenthesis) for number of fixations toward pictorial health warnings and for self-reported scores for avoidance, reactance, susceptibility, effectiveness and motivation by health risk presented on the label.
